# Enhancing Early Engagement (E3) in mental health services training for children’s advocacy center’s victim advocates: feasibility protocol for a randomized controlled trial

**DOI:** 10.1186/s40814-021-00949-2

**Published:** 2021-12-06

**Authors:** Erin K. Taylor, Alex R. Dopp, Kaitlin Lounsbury, Yutian Thompson, Michelle Miller, Ashley Jorgensen, Jane F. Silovsky

**Affiliations:** 1 Center on Child Abuse and Neglect, Department of Pediatrics, University of Oklahoma Health Sciences Center, 940 NE 13th St #4900, Oklahoma City, OK 73104 USA; 2 RAND Corporation, 1776 Main Street, Santa Monica, CA 90401 USA; 3 National Children’s Alliance, 516 C St NE #100, Washington, DC 20002 USA; 4 HumRRO, 700 North Hurstbourne Parkway, Suite 100, Louisville, KY 40222 USA

**Keywords:** Child maltreatment, Service navigation, Children’s Advocacy Centers, Training, Consultation

## Abstract

**Background:**

Child maltreatment is a major public issue in the United States, yet most children affected by abuse or neglect never engage in evidence-based practices (EBP) for child mental health. Children’s Advocacy Centers (CACs’) are uniquely situated to serve as Family Navigators who connect children impacted by maltreatment to appropriate EBPs. In fact, the CAC position of Victim Advocate mirrors the Mental Health Family Navigator national initiative.

**Methods:**

The feasibility study protocol is to develop, implement, and evaluate web-based and consultative training for Victim Advocates to enhance early engagement in services (E3 training). The interactive web-based training embeds key targets of knowledge and skills related to family engagement, trauma, and EBP services. Participating CACs were randomized to E3 webinar-based training, E3 webinar plus consultation, or delayed training. The project will test the E3 training’s impact on key mechanisms of change (e.g., knowledge, skills) to improve rates of screening, referral, and access to EBP services. The feasibility of implementing the training program and differential impact and costs by level of training will be examined.

**Discussion:**

The overarching goal of this project is to test the feasibility of training that is readily implemented through CACs and examine the mechanisms for improving early engagement and, ultimately, child, and adolescent mental health outcomes. Results and cost findings will be used to plan a large-scale comprehensive, mixed-methods hybrid type II effectiveness-implementation and cost-effectiveness trial of family navigator E3 training. If outcomes are positive, considerable infrastructure exists to support the scale-up and sustainability of E3 training nationwide, by embedding the training in national CAC training protocols.

**Trial registration:**

NCT04221633

**Date and version identifier:**

March 25, 2021; Vers. 1.0 (original); September 11, 2021; Vers 2.0 (revision); October 29, 2021; Vers. 3.0 (revision)

Contributions to the literature

- Children affected by child abuse and neglect are at high risk for psychological sequela and yet rarely receive evidence-based mental health treatments, even when such practices are available.
- Few training and implementation strategies have been developed to address these challenges.
- The Enhance Early Engagement (E3) training is designed to improve Children’s Advocacy Center’s Victim Advocate’s knowledge and skills at screening for mental health concerns, referral, and engagement in evidence-based practices.
- This protocol describes a randomized pilot trial of feasibility, implementation outcomes (knowledge and skills), mechanisms of effect, and costs across three conditions.
- Results will advance the science behind implementation strategies designed to increase engagement in evidence-based practices and will help prioritize strategies for additional testing and eventual scale-up.

## Background

Child maltreatment is a major public health issue in the United States (U.S.), with over 650,000 children and adolescents confirmed as victims of abuse and neglect in 2019 [1]. The effects of maltreatment and trauma on youth are wide-ranging, with both immediate and long-term impacts, including posttraumatic stress disorders [2], disruptive behavior conditions [3, 4], depression [5], and problematic sexual behavior [6]. These problems can persist into adulthood and lead to negative well-being, educational, and occupational outcomes, with an annual economic burden reaching $120 billion [7, 8]. A variety of evidence-based practices (EBP) have been found to be more efficacious than treatment-as-usual in reducing symptoms for mental health conditions associated with child maltreatment [9]. Unfortunately, the ability of EBP to mitigate the impact of child maltreatment is encumbered because most children in need never engage in EBP [10–15]. In fact, families that most need intervention often is the most difficult to engage successfully due to the barriers they face [15].

We could transform this current state of affairs by capitalizing on an existing national network of Children’s Advocacy Centers (CACs). CACs were created in the mid-1980s to provide coordinated responses to child maltreatment by integrating social, legal, medical, and mental health responses and interventions for child abuse and neglect [16]. Notably, over 370,000 children were served at 900 CACs nationwide in 2019 [17]. Victim Advocates at CACs play a critical role for children, as they are tasked with guiding the family through the entire CAC process, including facilitating access to EBP. National data suggests that Victim Advocates already refer children seen at CACs to mental health services at high rates [18]. Unfortunately, approximately 40% of those families who receive a mental health referral from CACs never attended an initial appointment [18].

The National Children’s Alliance (NCA) is the membership organization and accrediting body for CACs. Through nationwide initiatives over the past decade, NCA has prioritized efforts to improve the engagement of children and caregivers in mental health screening and EBP treatment [19]. EBP may be provided to families directly at the CAC or via linkages to providers at community agencies. Victim Advocates are in a prime position to facilitate early engagement in EBP to improve child mental health outcomes. Unfortunately, it is not common practice for all Victim Advocates to receive systematic training in a mental health screening or engaging children and families in EBP, resulting in significant missed opportunities to streamline families’ access to high-quality mental health care. Previous research has found that system navigators with expertise in mental health care are able to connect individuals to evidence-based services successfully and help to maintain their engagement through successful completion of services [20, 21]. As such, the next step needed is to examine whether the application of system navigator knowledge and skills training for Victim Advocates will improve the family’s engagement in EBP.

The current project protocol is to develop, implement, and evaluate the feasibility of a two-level, evidence-informed protocol to train Victim Advocates in (a) mental health screening, (b) family engagement strategies, and (c) EBP identification and referral. The Victim Advocate Enhancing Early Engagement training (E3 training) will seek to improve the short-term mental health outcomes of children affected by maltreatment by improving rates of mental health screening and EBP engagement.

### Mental health screening

There is no existing consistent, structured CAC protocol for Victim Advocates to identify and refer children for mental health services. Furthermore, Victim Advocates are generally bachelor’s level professionals who do not have formal training in mental health diagnoses and treatment. Previous work has found that child welfare workers with limited child mental health knowledge were able to successfully screen over and subsequently refer, as appropriate, 17,000 children in foster care after receiving brief training in mental health screening [22]. Additionally, Victim Advocates have reported greater rates of confidence in mental health referral decisions when using a mental health screener [23]. Thus, we propose that the E3 training will improve Victim Advocates’ identification of children’s mental health needs by implementing a consistent process for screening.

### Family Engagement Strategies

Even when Victim Advocates are able to screen and identify the need for child mental health services, they face multiple challenges in successfully linking families to EBP. Low engagement rates of families in mental health treatment are common [23, 24]. Research has suggested that less than half of Medicaid-eligible families in urban outpatient settings attend an initial appointment [24], and over two thirds drop out within 7 sessions [25]. Engagement of families in interventions for child maltreatment is wrought with challenges, as families face numerous barriers, including competing demands, stigma associated with mental health services, discrimination, social reactance to court-ordered treatment, logistical barriers (e.g., transportation, child care), and other factors that impact disparities and engagement [24, 26–28]. Thus, engagement is complex, involving individual, familial, provider, agency, and community factors [29]. What appears to make a difference in engagement, despite these challenges, is targeting caregiver perceptions of mental health treatment, while implementing strategies to reduce barriers and promote access, providing education about EBP, and supporting goal-setting [29]. To this end, E3 training will target these areas by teaching Motivational Interviewing (MI) [30] and the Training Intervention for the Engagement of Families program (TIES) skills [20].

Both MI and TIES target barriers to engagement in mental health services. MI focuses on addressing a participants’ willingness to change and improving intrinsic motivation. Originally developed to address substance abuse, MI has been applied to behavioral change in multiple service sectors. Research has suggested MI can be successfully taught to “lay individuals” (i.e., non-professionals) through multiple modalities, including webinars [31, 32]. Relatedly, TIES addresses perceptual, historical, and external barriers that families face to engaging in mental health services through listening, relationship building, and problem-solving. TIES embeds MI in the approach, and both focus on collaborative, empathetic interactions with families from the initial encounter [20]. Both MI and TIES have demonstrated a marked increase in initial and long-term engagement in mental health services [25, 33–35].

### EBP identification and referral

Previous research has demonstrated that combining engagement strategies and EBP leads to significantly improved retention in services and positive outcomes in families involved in child welfare [36–38]. The availability of EBP has increased over the past several decades [39], but they are not yet standard in most communities [40]. Further, it can be challenging for those not trained in mental health to understand what qualifies as EBP and how to identify EBP providers in their own communities, as CAC directors have expressed a need for themselves and their CAC staff to receive training on evidence-based assessment and EBP [41]. The mental health division of NCA has examined, identified, and supported the training of EBP for child maltreatment-related symptoms and concerns for all accredited CACs to address this need. However, without a consistently applied protocol for identifying and engaging families in EBPs, children seen through CACs nationwide are less likely to receive needed EBP. To this end, we propose that E3 Training on child mental health, EBP for targeted needs, and identifying EBP in their community will improve families’ linkage to EBP by providing Victim Advocates with an evidence-informed approach to identifying and engaging families in services that are most likely to benefit them.

### Overview of study design

The three components of the E3 training (i.e., screening, engagement, referral to EBP) targets Victim Advocates’ skills and knowledge, improving their efficacy in engaging families in mental health services, while also increasing the likelihood that children are referred through CACs will receive the best available treatments. Therefore, the pilot project was designed to examine whether the expansion of Victim Advocates’ activities of screening and referral by supporting engagement messaging by the Multidisciplinary Team (MDT), identifying EBP in the community, and implementing strategies to overcome barriers was feasible. Further, the pilot is designed to examine the feasibility of the methods for recruitment, training, and data collection. Developing these skills in Victim Advocates is proposed to help children and adolescents with maltreatment histories engage in EBP to address the potential impact of their traumatic experiences in the future. If outcomes are positive, considerable infrastructure exists to support the distribution and sustainability of E3 training, as the training can be readily accessed and embedded in CACs under the guidance of NCA training protocols.

The primary goal of this pilot study is to examine the initial feasibility of training and study methods implementation, as well as outcomes and comparative costs of the E3 training. With regard to feasibility, CACs and Victim Advocates do not consistently participate in large-scale research projects; for many, the E3 project would be the first time they participated in structured data collection and submission. It was important to pilot the project in order to determine whether they would be able to participate successfully and what the necessary supports were in order for CACs to complete research benchmarks at pre, post, and follow-up. Further, we wished to examine data collection methods to determine best strategies for a large-scale trial. We also sought to examine whether the training could be successfully implemented with Victim advocates, including participation in all webinars and consultation calls. Most Victim Advocates have limited experience engaging in consultation, so the pilot will allow the research team to examine the success of consultation with Vitim Advocates and to determine necessary modifications for future projects. Overall, feasibility outcomes include attendance in training activities, data submission and completion, and acceptability and satisfaction of Victim Advocates and leadership with the E3 training.

Further, the protocol also evaluates the implementation of a two-level approach to the E3 training: E3w, a professionally developed webinar-only training, compared to E3w+c, the webinar training augmented with 10 consultation calls with experts in mental health and family engagement. Notably, webinars are cost-efficient compared to in-person training, as they primarily require initial expenditures for their creation and incur only limited additional costs for ongoing maintenance and participation. Webinars can also be made broadly available, even in remote settings, and require limited disruption to direct service time when compared to the time needed for travel and in-person training. Alternatively, consultation models are inherently more expensive, as they require ongoing staff to lead consultation and require consultants and consultees to devote the time that could otherwise be utilized for other valued professional activities (e.g., engaging in client meetings). However, consultation allows for directed practice of skills and continued learning via guided discussions and role plays. Previous research on EBP adoption has suggested that ongoing consultation improves training outcomes [42]. In addition, the practice of skills is crucial for behavior change in mental health settings [43]. Finally, telemedicine technology, similar to what was used for the consultation calls, has successfully been utilized to deliver coaching and direct feedback for mental health and associated professionals at a lower cost than in-person training while also improving outcomes over the initial training alone [44]. Given the various trade-offs between the E3w and E3w+c approaches, it is critical to pilot both conditions in order to compare them directly prior to selecting a candidate training strategy for potential national scale-up of E3 training.

## Methods/design

### Study design overview

**Fig. 1:**
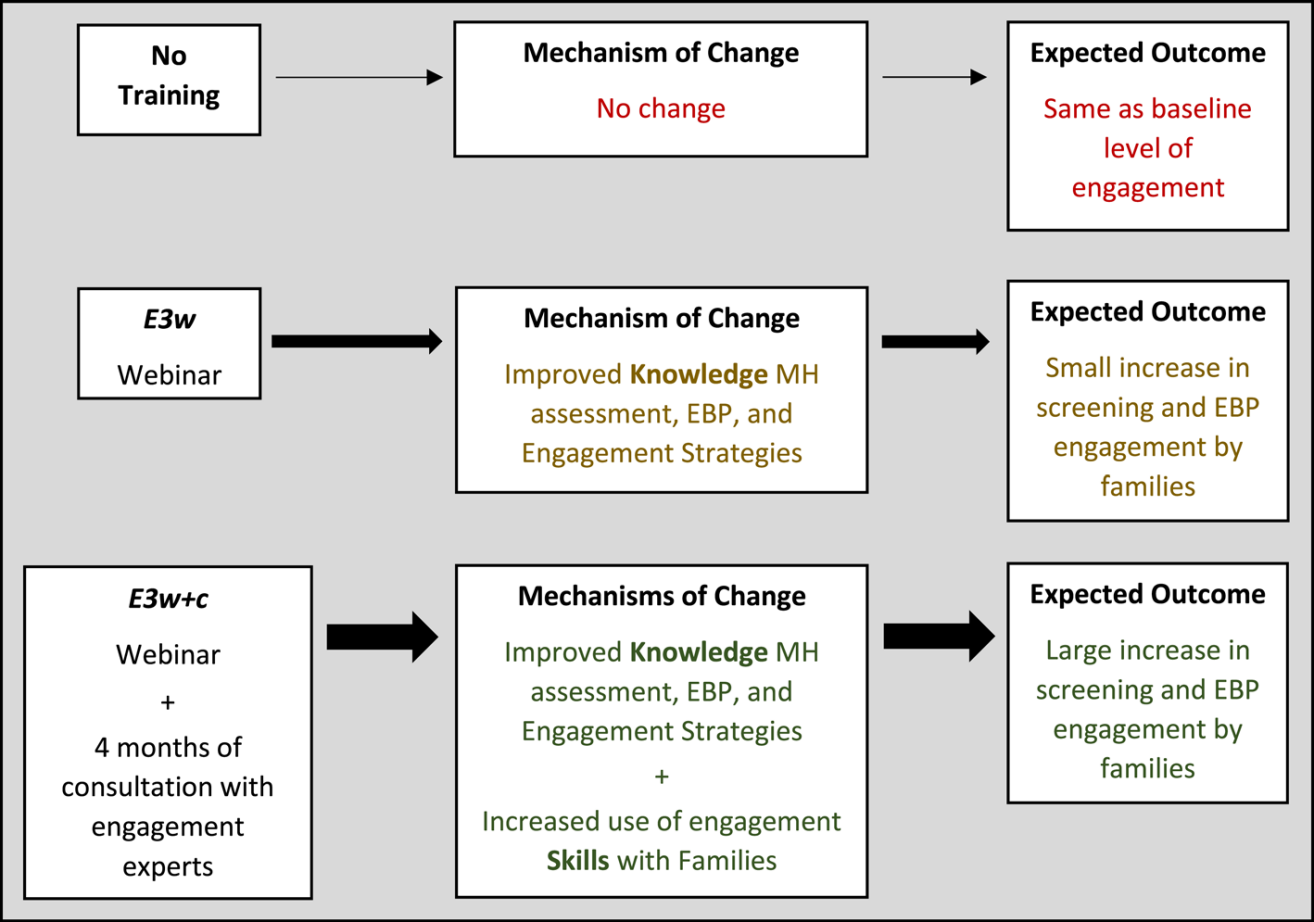
Flow chart of study premise and hypotheses

For this randomized controlled trial (RCT), we are testing the feasibility, outcomes, and cost of the two levels of E3 training compared to current practices in CACs in a 1:1:1 allocation ratio. The protocol is to randomize the CACs to the E3w, E3w+c, or a delayed waitlist control condition, with data collected directly from training participants pre-training, immediately post-training, and at follow-up. Further, the protocol is designed to utilize NCA’s standard data systems to collect outcome data from caregivers and community stakeholders pre- and post-training. We hypothesized that the E3 training would be readily implemented within the training structure of NCA and that Victim Advocates and CAC Directors would report high levels of satisfaction with the training. More importantly, we hypothesized that E3w alone would improve Victim Advocates’ knowledge, resulting in a minor improvement in EBP engagement, while the addition of consultation in E3w+c would lead to increased use of engagement skills, thereby resulting in greater improvement in family engagement in EBP (see Fig. 1 for flow diagram). For purposes of the current study, we will examine family engagement via rates of mental health screening, rates of referral to EBP by Victim Advocates, and family attendance at the first session. Cost data was included in the protocol to examine cost-effectiveness in future studies.

At the time of this manuscript, the training was developed and implemented, and all data was collected. Below we describe the training, measures, and implementation of the E3 study that was conducted.

### Webinar development

Training for both E3w and E3w+c was provided via a web-based platform. Although webinars themselves are not unique to the training of professionals in mental health or child maltreatment, by using recommended practices for webinars (e.g., pre-work activities, interactive components, provision of follow-up resources [45]), we are testing an interactive and engaging training. In addition, consistent with previous research indicating the need for CAC leadership involvement [41], a web-based training session was provided to CAC administrators and community stakeholders (i.e., MDT members) across both E3 and E3+w conditions. The goal of the MDT webinar was to provide education regarding the role of the Victim Advocate and strategies MDT members can use to enhance family engagement in EBP.

### Consultation plan

The E3w+c training involved two separate orientation training calls for Senior Leaders and Victim Advocates that reviewed the responsibilities and structure of the training. This was followed by 10 web-based consultation calls; Victim Advocates were required to attend 80% for successful completion, and attendance was taken at each call. Calls began weekly in order to solidify learning from the webinars; the final six meetings then took place biweekly. The meetings provided opportunities to individualize learning and practice skills related to mental health screening, engagement (TIES and MI strategies), and linkage to EBP. With Victim Advocates from multiple CACs on each call, there was an opportunity for shared learning, as each participant had the opportunity to share identified barriers encountered and gain feedback from experts and their peers.

### Site recruitment

**Fig. 2:**
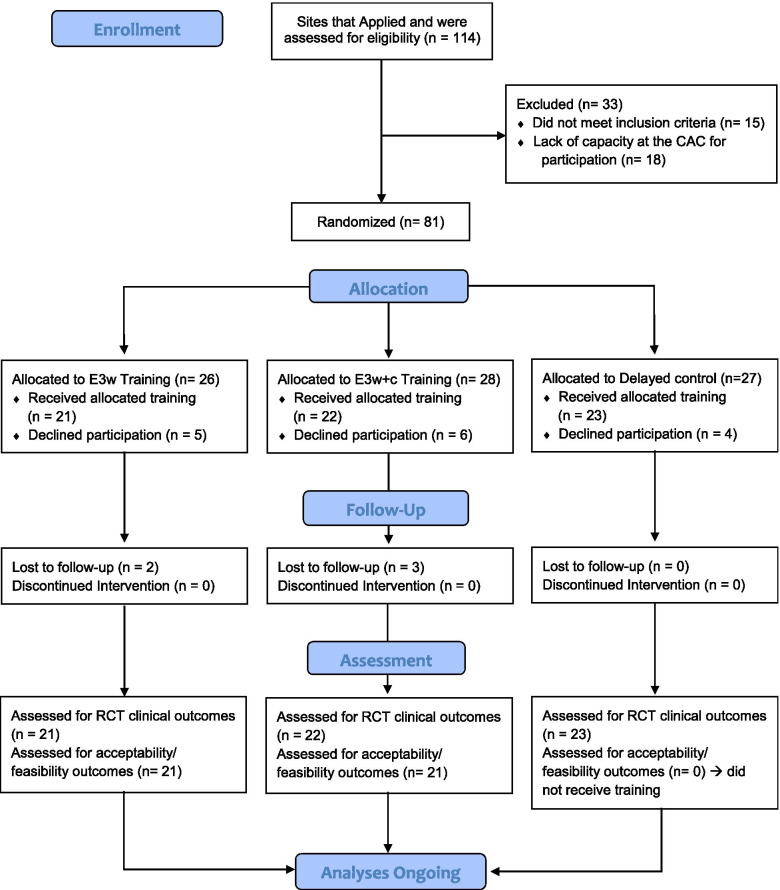
Flow diagram of site enrollment and randomization

Recruitment and selection of CACs took place in fall 2019. In order to examine the feasibility of the E3 training best, we sought to recruit a sample of CACs that reflected the variability of CACs across the U.S. Applications for training were released via email to all accredited CACs, and interested CACs completed an application to participate through NCA, with procedures following NCA’s established guidelines for the application, proposal evaluation, site selection, and implementation of training processes. Informational calls were held in the fall of 2019 to address questions and review the commitment required for participation in all aspects of the project (training and research). Applicants were reviewed for meeting the following inclusion criteria: (a) fully accredited by NCA, (b) either directly provided EBP for child mental health or had established and demonstrated linkages for services in the community, (c) participated in the NCA’s Outcome Measurement System (OMS), and (d) had Memorandum of Understanding (MOU) or data sharing agreements with all referral sources. The selection was made at the CAC site level, rather than the Victim Advocate level. This was to ensure that all Victim Advocates at the same CAC were placed in identical conditions, thereby avoiding any cross-sharing of knowledge across training conditions. See Fig. 2 for a flowchart regarding enrollment.

### Procedures

#### Timeline

**Fig. 3:**
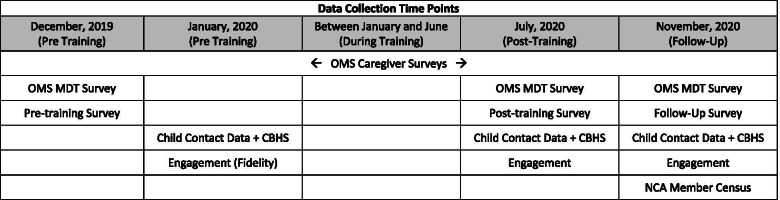
Data collection time points

CAC administrators (Senior Leaders) and Victim Advocates from the sites that met the inclusion criteria were invited to participate and complete consenting procedures, as approved by the University of Oklahoma Health Sciences Center. Informed consent was completed with all individual participants via an electronic platform (i.e., REDCap). Participants were informed that they are allowed to discontinue participation as a site or as an individual at any time. Multiple data collection methods were planned for pre-training, post-training, and follow-up (see Fig. 3). After each webinar, a short training evaluation form was provided to E3w and E3w+c participants. Data collection was monitored by the project coordinator, who assisted sites with any questions or concerns with support from the research team. A data monitoring committee was not utilized given the low level of risk for participating sites. Sites received $600 for their participation.

#### Data sources

##### The Outcome Measurement System (OMS)

Measures completed by caregivers and MDT members were captured through three of the NCA Outcome Measurement System’s surveys: (a) the Initial Visit Caregiver Survey, offered at the end of a CAC visit; (b) Caregiver Follow-Up Survey, completed approximately 6 weeks after the family’s initial visit to the CAC; and (c) the MDT survey, completed twice over the study. CACs were required to participate in all three OMS survey systems. Anonymous and voluntary, the surveys are delivered via both paper and electronic methods either on-site or through take-home options. The survey questions are a mix of Likert-scale, yes/no, and open-ended items to provide a variety of ways respondents could share opinions, concerns, and suggestions. The standard OMS surveys were modified for the current study in order to include questions assessing Victim Advocate family engagement skills and connection to EBP.

##### NCA Member Statistics & Census

CACs provide administrative data to NCA on the scope of services provided and remaining service needs through two statistical sources: NCA statistics submitted every 6 months through the NCATrak case management system and NCA Member Census Surveys collected every 2 years through Qualtrics. Statistics include basic outputs like the number of children served, client demographics, and case resolutions. The Census Survey includes more detailed questions on topics like funding sources, staffing information, and information on mental health services provided by CACs and partner agencies. The most recently available Census was collected in the summer of 2020.

##### REDCap

All project-specific data, including measures noted below, client tracking information, and any other assessments completed by the Victim Advocates and Senior Leaders were collected via REDCap at the University of Oklahoma Health Sciences Center [46, 47]. Victim Advocates at each site were able to enter data at any time and were only able to view their own site’s data.

##### Pre-RCT survey

In the first year of the project, prior to the selection and randomization of sites, we implemented an electronic survey of Victim Advocates and Senior Leaders across all CACs. Collected through NCA’s Qualtrics system, the survey was distributed to the national network of CACs. Questions focused on the current roles, responsibilities, activities, tools, and management of Victim Advocates. We received responses from 915 Victim Advocates and 540 CAC Directors, which were then utilized by the training team to develop the E3 training. In addition, several items on mental health screening procedures and barriers that Victim Advocates face when engaging families in EBP were used in the adaptive randomization procedure (see below).

### Measures

Multiple measures were collected over the course of the study. Details regarding these measures are described in Table 1.

**Table 1:** Assessment measures across participants and time points

Construct	Measure	Brief description	Time points	Participants	Method/procedure
*Proposed mechanisms of change*					
Knowledge	Test of knowledge	39-items developed to assess advocates knowledge of screening, barriers, engagement strategies, and trauma; mixed format of multiple choice and true/false	Pre-training, post-training, follow-up	Victim Advocate	Self-Report via REDCap
	Self-evaluation of knowledge	20-item measure assessing self-perceived knowledge of evidence-based treatment, mental health, screening, trauma, and engagement strategies	Pre-training, post-training, follow-up	Victim Advocate	Self-Report via REDCap
Skills	Fidelity	Items developed for respondents to rate use of engagement skills during a meeting with caregiver	Pre-training, post-training, follow-up	Victim Advocate	Self-Report via REDCap
*Potential moderators*					
Attitudes	Victim Advocate Attitudes	22-items developed to assess respondents’ attitudes towards mental health, screening, trauma, and supervision	Pre-training, post-training, follow-up	Victim Advocate	Self-Report via REDCap
Barriers to treatment	Perceived barriers to treatment	Advocates perceptions of barriers families face when attempting to access evidence-based mental health services	Pre-training, post-training, follow-up	Victim Advocate	Self-Report via REDCap
Ethnic Sensitivity	Ethnic sensitivity inventory: precontact [48]	5-items assessing respondent’s sensitivity to other cultures and ethnicities	Pre-training, post-training, follow-up	Victim Advocate	Self-Report via REDCap
Organizational support	Perceived organizational support [49]	16 items assessing respondents’ perceptions of the support they receive from their agency	Pre-training, post-training, follow-up	Victim Advocate	Self-Report via REDCap
Supervisory support	Perceived supervisory support [50]	16 items assessing respondents’ perceptions of the support they receive from their supervisor	Pre-training, post-training, follow-up	Victim Advocate	Self-Report via REDCap
Self-Efficacy	General self-efficacy [51]	8-item measure assessing belief in one’s capability to meet task demands across contexts (i.e., self-efficacy)	Pre-training, post-training	Victim Advocate	Self-Report via REDCap
Executive functioning	Behavior Rating Inventory of Executive Function-Adult [52]	Assessment of executive functioning abilities (abbreviated version)	Pre-training	Victim Advocate	Self-Report via REDCap
Learning anxiety	Learner Anxiety Assessment	3-item measure assessing concerns regarding learning new information	Pre-training	Victim Advocate	Self-Report via REDCap
Motivation to learn	Motivation to learn —general [adapted from [53, 54]	3 items assessing trait-like motivation to learn new things	Pre-training	Victim Advocate	Self-Report via REDCap
	Motivation to learn —specific [adapted from [53, 54]	5-items assessing motivation to learn information specific to the E3 training	Pre-training	Victim Advocate	Self-Report via REDCap
*Training evaluation*					
Webinar evaluation	Post-webinar evaluation	Brief measure assessing satisfaction with each webinar and feedback regarding potential improvements	Completed after each webinar	Victim Advocate	Self-Report via REDCap
Overall training evaluation	Post-training evaluation	Measure assessing overall satisfaction with, acceptability of, and feedback regarding E3 training, including webinars, consult calls (when applicable), and related assignments	Post-training	Victim Advocate	Self-Report via REDCap
				Senior Leader	
*Participant and agency characteristics*					
Demographics	Demographic Questionnaire	Basic demographic information	Pre-training, post-training, follow-up	Victim Advocate	Self-Report via REDCap
				Senior Leader	
CAC agency level	Agency Level Questionnaire	Information specific to the CAC, including annual numbers of forensic interviews, funding structure, etc.	Pre-training, post-training, follow-up	Senior Leader	Self-Report via REDCap
	NCA Census	Survey completed by CACs every other year assessing funding sources, staffing information, and detailed information on mental health services provided by CACs and partner agencies	2020	Senior Leader	NCA Census Survey (Qualtrics)
Multidisciplinary team	OMS MDT Biannual Survey	Anonymous, voluntary survey completed by members of the MDT regarding the functioning of MDT, feedback on the process, and suggestions for improving the MDT	Twice yearly survey	MDT members	OMS MDT Survey
*Outcomes*					
CAC Outcome data	Child Contact Data	Basic demographic information about the child, details regarding who met with the family, what was provided during the visit, and whether the family was (1) referred to and (2) subsequently attended a mental health appointment.	Pre-training, post-training, follow-up	Victim Advocate	REDCap
	OMS Initial Caregiver Survey	Anonymous, voluntary survey typically collected on-site at the end of the CAC visit via paper surveys or electronically; obtains family feedback regarding their visit and the support and follow-up provided by CAC staff	Throughout the year, as per CAC protocol	Caregiver report	OMS Initial Caregiver Survey
	OMS Follow-up Caregiver Survey	Anonymous, voluntary survey typically completed via a phone call 4 to 8 weeks post-visit; collects additional feedback regarding on-site visit, support, and subsequent follow-up by CAC and MDT staff	Throughout the year, as per CAC protocol	Caregiver report	OMS Follow-up Caregiver Survey
*Screening*					
Mental health screening	Child Behavioral Health Screener	Pediatric Symptom Checklist (PSC-17 [55];) plus 3 items focused on clinical-level child trauma symptoms, 6 items assessing functional impairment, and 3 critical items assessing suicidality, problematic sexual behavior, and substance use.	Pre-training, post-training, follow-up	Caregiver report (total score)	REDCap

#### Proposed mechanisms of change

The key mechanisms proposed to impact rates of child mental health screening, referral, and linkage to EBP via E3 training are changes in Victim Advocates’ *knowledge* and family engagement *skills*.

A self-report knowledge test directly examined the knowledge Victim Advocates gain through the training process. Items developed focused on engagement strategies, trauma and effects of trauma, evidence-based mental health treatments, screening for child mental health concerns, and strategies for identifying EBP in their own communities. Our goal was to test change in knowledge acquisition by Victim Advocates.

Skill (i.e., fidelity) measures were adapted from previous research examining self-reported fidelity to the TIES model [56], as well as current coding manuals for MI fidelity [57, 58] to create a self-report checklist of skills taught in the training. In consultation with the TIES experts, we developed a self-report checklist that includes both engagement-consistent behaviors (e.g., inquiring about previous mental health experiences) as well as behaviors counter to the MI and TIES strategies (e.g., providing unsolicited advice). The inclusion of both item types ideally decreased the demand for overly positive responses by Victim Advocates. Such a measure will allow us to examine skill development and its influence on primary outcomes (see below).

#### Clinical trial outcomes

Targeted outcomes are as follows: implementation of screening, referrals for services, successful linkage to at least one mental health appointment, types of services accessed (i.e., EBP status), and reduced caregiver stress. These were captured via both OMS caregiver surveys and through REDCap surveys completed by the Victim Advocate to address (a) screening forms implemented, (b) engagement strategies used, (c) results of screening, (d) referrals made, and (e) first treatment session documented by date.

#### Feasibility outcomes

As a pilot study, we included multiple measures of feasibility. Specifically, we developed training evaluation forms to be completed by Victim Advocates immediately after each webinar to determine didactic training acceptability. We implemented an overall training evaluation form, completed by both the training groups at the post-training data collection wave. The overall evaluation measured Victim Advocate and Senior Leader satisfaction with E3 training, their perceptions of the acceptability of the training method for their CAC, and any feedback they had regarding improving the training process in the future. These results, in conjunction with observations of rates of successful data completion and attendance at required training activities, will be examined when determining whether the E3 training is feasible for CACs and any necessary adjustments for future trials. Examination of the feasibility of the study methods was planned through evaluation of the success of data collection completion and cleanliness, as well as feedback from and support provided to the Victim Advocates and Senior Leaders addressing data collection procedures.

#### Moderators

Victim Advocates will approach the training with varying levels of goals, motivations, learning styles, and preferences. In addition, characteristics of the CAC may impact the uptake of the training. As such, surveys of the Victim Advocate and the CAC Director were designed to capture these potential moderators of outcomes. The Victim Advocates completed surveys addressing attitudes, motivation to learn, learning anxiety, ethnic sensitivity, organizational and supervisory support, and barriers to services (see Table 1), while CAC Directors provided descriptive data of the CAC personnel and activities.

#### Costs

To capture direct and indirect costs associated with implementing the E3 training, during the project we tracked (a) the amount of time Victim Advocates spend completing the webinar and pre-work activities, (b) the number and length of consultation calls attended by each Victim Advocate (if applicable), and (c) the number of screening assessments and referrals completed at each CAC. The detailed cost information was collected at the follow-up, comprising of questions about salary/wages and benefits, time, and resource use. Costs associated with the development of the training materials and resources were also collected from the E3 training team to examine overall training development costs.

### Randomization

Prior to study implementation, a power analysis was conducted to determine how many sites would be needed per randomized condition. Because we had three treatment conditions (i.e., E3w, E3w+c, delayed waitlist control), we planned to assess intervention effects for all three two-way combinations of interventions. The power analyses were conducted for each of these two-way comparisons. To avoid overestimating power [59], we used the smallest number of clusters in an intervention group to estimate power. Power analyses were conducted using the Optimal Design software [60]. With a small intraclass correlation (*ρ*=0.05) and 50 total CACs (i.e., total clusters across a pair of intervention conditions), the minimal detectable effect size (MDE) is relatively small *δ*=0.19 as a standardized mean difference assuming 80% power and a type I error rate of 5%. This also assumes there are at least 200 referrals per CAC. For the same design criteria and a larger intraclass correlation (*ρ*=0.50), the MDE is large at 0.57. Overall, power analyses suggested that we randomize at least 25 CACs per condition. We received 114 applications, all of which were evaluated for inclusion criteria. In addition, the research team determined if the CAC lacked the capacity to participate in the training (e.g., only one part-time advocate employed, ongoing participation in multiple training initiatives), they were not included in the randomization. After review, 81 sites were eligible to participate, and all were randomized.

The adaptive randomization process began with a preliminary exploration of baseline covariates that were correlated with the outcome variable caregiver engagement. Variables were taken from pre-existing data collected through the NCA Census (*N* = 753, *q*^1^ = 222), NCA statistical data [*N* = 838, *q* = 68], OMS Surveys (including the caregiver follow-up survey [*N* = 490, *q* = 16] and MDT survey [*N* = 560, *q* = 14]), and pre-RCT surveys (CAC director survey [*N* = 540, *q* = 123] and advocate survey [*N* = 880, *q* = 156]).

Based on factors hypothesized to influence the outcome of interest (i.e., child engagement in EBP), the initial analysis included the following variables: (a) type of location (urban vs. rural), (b) region of the CAC (e.g., Northeast, Southern), (c) number of children served, (g) number of total CAC staff, (d) number of advocates on staff, (e) organization type (e.g., hospital-based, government-based), (f) EBP services provided onsite or via community, (h) level of MDT collaboration, (i) number of barriers CAC staff report experiencing when referring families to EBP, (j) use of a mental health screening tool, (k) advocates previous training experiences, (l) number of children reported to EBP, and (m) number of children who received EBP. The main purpose of the exploratory analysis was to specify the factors most predictive and apply them as the baseline covariates. Both variables (l) and (m) were used as outcomes, and the rest of the variables were predictors in generalized linear models. Because of the exploratory nature of this aim, as well as the existence of missing data, the major risk was a false discovery due to capitalizing on chance. Therefore, the analysis practiced the stepwise model selection based on multiple imputed data [61]. Notably, variables (a) region of the CAC, (b) number of barriers when referring families to EBP, and (c) use of mental health screen tools appeared in more than 50% of the selected models from twenty imputed data. Therefore, this analysis used these three variables as the covariates in the adaptive randomization.

Covariate adaptive randomization is an approach to ensure that the participants are approximately balanced with respect to covariates in the randomization [62]. The current analysis utilized the method of permuted block randomization with eight strata (4 region areas × 2 screen tool usage levels) to assign 81 CAC sites randomly into three arms. Group A (*N* = 26), Group B (*N* = 28), and Group C (*N* = 27), corresponding to E3w, E3w+c, and delayed control, respectively. A preliminary baseline equivalence test was also applied to check whether any differences between the three arms existed. It did not find any difference between groups on children’s rate of referral to EBP (*F*_(2, 78)_ = 0.185, *p* = 0.832), rate of EBP receipt (*F*_(2, 78)_ = 0.146, *p* = 0.864), or number of advocates on staff (*F*_(2, 78)_ = 1.423, *p* = 0.247).

### Proposed analyses

Evaluation and analysis of all data collected are ongoing.

#### Quantitative analytic plan

The outcomes analysis will be conducted from the post-training and follow-up assessments of Victim Advocates and Senior Leaders, as well as the continual collection of OMS survey data from caregivers and team members. The variables collected from Victim Advocates and Senior Leaders are the time-varying and CAC-varying *provider fidelity*, *knowledge*, and *perceptions* of the utility of training. In addition, outcomes will include *family engagement*. Statistical analysis will include, but is not limited to, the following: (a) applying linear mixed-effect models to evaluate the changes of the primary outcomes between conditions across time, should the distribution of the outcomes and residuals suggest being appropriate [63, 64]; and (b) investigating the mechanism that is responsible for the causal effect between training conditions and outcomes, with the mediator of knowledge/skill achievement. Covariates collected (e.g., perceived supervisory support, learning anxiety) will also be examined for their influence on the outcome of interest. As a feasibility study, the principal goal at this stage is to conduct an initial examination of training implementation and whether the Victim Advocate knowledge and skills change due to training, what factors might be associated with the change, and how that influences family engagement in mental health services. Further, descriptive statistics will be calculated to examine Victim Advocates ratings of acceptability and satisfaction with the training, as well as rates of attendance in training and consultation.

Missing data will not be avoidable due to the large amount of data collected from sites across the nation, and the repeated measurements across multiple time points. We will examine rates of missing data, as well as data cleanliness, to determine Victim Advocates abilities to complete data submission successfully. Further, stochastic multiple imputation methods will be used to handle missingness, if the assumption of ignorable missing mechanism can be held [65, 66]. In addition, analyses will be “intent-to-treat,” such that individual participants or sites who leave the study will be included in analyses.

All the analysis will be completed by the statistical package R (3.5.2) [67] with multiple packages, such as the dplyr, tidyr, ggplot2, lme4, stats, readr, and mice.

#### Qualitative analytic strategy

The research team plans to conduct thematic analysis of all qualitative responses on evaluation, feasibility outcomes of acceptability and satisfaction, and clinical trial measures. To do so, all responses to each qualitative question will be reviewed in their entirety in order to identify broad themes within the responses. Themes will be organized into a broad codebook, and additional coding will focus on refining themes further. Coding will be conducted by multiple members of the research team, and interrater reliability will be determined through cross-coding of responses and comparison of identified themes. Discrepancies will be reviewed with the larger research team to discuss and finalize coding.

#### Cost analysis

We will generate descriptive statistics from the quantitative cost data to describe typical costs (i.e., means) and variability in costs (i.e., standard deviations) associated with the delivery of the E3 training. Direct costs will be calculated in terms of the cost of the resource and the frequency of its use (e.g., consultation fee x number of consultation sessions). Indirect costs will be calculated by applying a shadow price [68], which estimates the value of lost productivity for alternative professional activities of CAC staff, to time spent on training activities (i.e., hourly shadow price x hours of training activities). All cost estimates will be placed on the same metric through the adjustment to (a) an index year using the Consumer Price Index [69] to account for inflation and (b) national average U.S. dollar values using the Council for Community and Economic Research Cost of Living Index [70] to account for costs of living differences between CAC locations. We will sum all direct and indirect expenses separately before the calculation of descriptive statistics and examine descriptive statistics for total (i.e., direct plus indirect) costs.

## Discussion

### Innovation and anticipated contribution

Child maltreatment and co-occurring traumas (e.g., domestic violence, parental substance abuse) are core adverse childhood experiences. Mounting research has demonstrated the immediate and longstanding impact of such experiences on physical and mental well-being [71–73]. Effective mitigation of this negative impact involves timely engagement in EBPs that have demonstrated positive effects on well-being [74, 75]. By providing linkages to services upon the outcry of child maltreatment, service navigators within CACs can facilitate both the immediate connection to needed mental health treatment as well as address “deep-rooted issues related to distrust in providers and the health system that often lead to avoidance of health problems and non-compliance with treatment recommendations” ([76] p. 3543).

The success of previous trials with service navigators within health care settings [76–79] were built upon for the current feasibility study. Uniquely, the service navigator model tested in this project occurs within the CAC, the location of child forensic interviews, and other key interventions provided as part of the investigation of child maltreatment. These multidisciplinary settings are located across the country and connected through the network established by NCA. A nationwide impact on access to EBP for children who experience child maltreatment is feasible given the reach of the 900 CACs across the country [80]. Broadly, no other known existing national system of programs can add one additional component to their existing services (i.e., training Victim Advocates in mental health screening, engagement, and EBP referrals) and have such a widespread impact on the mental health outcomes of children. Given the great potential for change, it was important to pilot training of Victim Advocates to determine whether E3 training could be scaled up, examined in a large-scale trial with child outcomes, and made available to all CACs and Victim Advocates nationwide.

The approach of directly addressing disparities in mental health care is another distinction of this trial. Research has suggested that there are significant socioeconomic disparities in the experience of child maltreatment, such that children living in disadvantaged neighborhoods and poverty are disproportionately affected by abuse and neglect [81]. NCA Accreditation Standards require that CACs provide services to clients regardless of their ability to pay, ensuring that all children receive necessary services. In addition, although previous work has suggested that maltreatment may occur at higher rates in minority populations, this effect is largely due to the poverty and marginalization experienced by certain racial groups [82]. As youth who are not White have been found to be less likely to engage in EBP for a variety of reasons, including experiences of discrimination and racism in the service sector [10, 13, 14], the development and implementation of the E3 training program may help to decrease racial and socioeconomic disparities in the delivery of EBP for children affected by maltreatment. TIES was selected to be a core of E3 training due to its success in enhancing engagement in services by families who are financially disadvantaged and impacted by service disparities [20, 24, 27].

Victim Advocates within CACs are primed for becoming service navigators given their location, role, and responsibilities. Considering the vulnerabilities of and service disparities experienced by the population served by CACs, successful early engagement in mental health services will need to rise above the baseline of providing education, case management, support, and assistance in accessing services. This trial tests the feasibility of integrating well-defined models (i.e., MI and TIES) designed to directly acknowledge and address distrust of service systems and integrate strategies found to reduce internal barriers to change behavior.

Large-scale implementation within complex service systems can be fraught with challenges. The expert recommendation is to construct small changes utilizing the current structure rather than attempt to overhaul the entire system [83]. This logic is readily applied to the current project, as the Victim Advocate position is already embedded and integral to the work of CACs. The E3 training is designed to enhance their capacity, testing whether enhanced skills of the Victim Advocate and the success of early engagement in EBP can be readily accomplished with web-based training alone (E3w) or significantly augmented through consultation (E3w+c). The results of this feasibility study will inform a larger randomized trial, integrating longitudinal design to examine the impact of Victim Advocate training on the child and family outcomes, as well as potential cost/benefit implications. If successful, the E3 project can inform the development of family navigator models for other settings (e.g., schools) [84] and clinical problems (e.g., problematic sexual behavior, for which service responses are even more challenging to coordinate than for maltreatment) [85]. Determinations of success will include the impact of the training on clinical trial outcomes, such that we see changes and improvements in mental health screening, use of engagement skills, and attendance at mental health sessions. Further, feasibility success will be determined by obtaining high rates of satisfaction and acceptability on training evaluations (ratings of 4 or 5 on Likert scale), high rates of attendance and completion of webinars and consultation (80% completion), and successful data submission.

### Limitations/practical and operational challenges

Although the current project offers many strengths, limitations and potential challenges warrant comment. Initial plans for data collection were to utilize NCATrak, NCA’s proprietary tracking software used by CACs to record data on their clients served, anticipating that most sites would be using the software. However, upon the investigation of the potential CAC sites’ procedures, the variety of database programs used, and the number of changes required for each system, the use of NCATrak was determined to be impractical for the collection and integration of data across CACs for the current project. As such, we shifted data collection to one central system, REDCap. However, the decision to use REDCap has not been without challenges, as we have had to train Victim Advocates on the system’s procedures and activities. Creating training videos on REDCap procedures and holding open “office hours” with the research team for troubleshooting concerns have facilitated the process. Future trials of the E3 training may benefit from additional data submission support for CACs, such as research technicians who can collect and enter data directly, in order to decrease the burden on Victim Advocates.

Further, to examine fidelity to the model, Victim Advocate implementation of engagement strategies like MI and TIES had to be collected via self-report methods, as observational methodologies were not within the scope of the feasibility project budget. Research has suggested that professionals are able to reliably self-report on their use of intervention techniques with a high level of concordance with an observer and expert ratings of the same variables [86, 87]. However, observational methodologies are planned to expand the examination and verify changes in skills in the next step of the research and larger-scale project.

A third limitation of the study is our ability to address all of the unique barriers faced by each family and CAC. The E3 training encouraged Victim Advocates to assess and address all of the potential barriers faced by their families, using evidence-based motivational strategies to improve their engagement in EBP. However, it is possible that the training’s focus on knowledge and skills of MI and TIES subsequently caused other important barriers to be missed. Future research may benefit from individualized assessment of the barriers within communities and additional training to Victim Advocates on best methods to tailor their responses to each family.

An unfortunate challenge that was unable to be addressed within the current study is the fact that mental health services, particularly EBP, are not readily available across all communities. The E3 project required that sites have linkage agreements with or provide on-site mental health services providing EBP, and Victim Advocates were trained to recognize EBP. However, it is possible that families were still unable to access quality services in their communities for myriad reasons. It is possible that only non-EBP were available in certain communities or for specific problem types. Alternatively, long waitlists may make access to services challenging, particularly for providers of color, who are significantly underrepresented in the mental health field. For BIPOC youth and families, being able to access services with a provider of color may have been important yet even more difficult to find. The challenges of EBP saturation and availability were beyond the scope of the E3 project but will have an impact on outcomes. As such, future research should consider direct measurement of these variables, as they are important potential mediators of outcomes.

The rate of job turnover of CAC personnel was also an unanticipated challenge in the current project. In response, we added measures related to turnover intent, job satisfaction, and burnout to the post and follow-up data collection waves to understand better this phenomenon within the CAC. Our experience highlights the importance of using a readily accessible, effective training platform, and the need to examine the impact of training on job retention. Previous research has demonstrated that training in EBP with associated consultation significantly improved job retention of bachelor’s level home-based parenting service providers working with vulnerable families (i.e., half the job turnover rate than the other conditions) [88]. Currently, we are implementing an intent-to-train analytic plan by asking those sites who left the training to complete data collection at post-training and follow-up. Lessons learned from this feasibility trial will inform a large randomized trial in the future.

Finally, another unanticipated difficulty has been COVID-19. The pandemic and related stay-at-home orders had a direct and profound impact on CAC activities as well as on the well-being of CAC staff and the families they serve. Transitioning CAC services to tele-health platforms was essential. Throughout the crisis, we endeavored to provide support to Victim Advocates in their understanding and skill in administering the screener and using TIES and MI strategies via the phone and telehealth platforms. By providing video training and additional consultation, we hoped to broaden the CACs’ use of the training. In addition, flexibility and changes in the timeline allowed CACs space to adjust to the changes caused by COVID-19 and improve learning. Finally, measures of COVID-19 impact on well-being and CAC functioning were integrated into post- and follow-up data collection waves, which will provide opportunities to examine the process of family engagement during considerable stress and strain.

### Future directions

CACs are uniquely well situated to connect families to EBPs in order to target the range of mental health symptoms and disorders of children impacted by maltreatment and other traumatic experiences. The overarching goal of this project is to test the feasibility of a Family Navigator training for Victim Advocates at CACs across the nation and to examine the mechanism that improves children’s early engagement in EBP. Ultimately, our goal is to improve child and adolescent mental health outcomes. Quantitative results will allow us to establish the efficacy of the training overall, while qualitative feedback provided by Victim Advocates, CAC directors, and MDT partners will allow us to determine what improvements and changes are needed to the E3 training to allow for wider implementation in the future. Finally, the results of the cost analysis will provide critical information about the resources required for E3 training and inform our approach to comparing economic costs and outcomes between different training models. If outcomes are positive, considerable infrastructure exists to support the scale-up and sustainability of E3 training, by embedding the training in all CACs under the guidance of NCA training protocols. Using the results of the current study, we plan to proceed to a larger-scale mixed-methods clinical effectiveness-implementation (Hybrid Type II [89]) and cost-effectiveness trial of the E3 training on child mental health outcomes. These various efforts will support examining the broader implementation of the E3 Family Navigator model through CACs nationwide, offering tremendous potential to reduce the social and economic impact of child maltreatment by linking some of our most vulnerable children and families to high-quality mental health treatment.

## Data Availability

The datasets used and/or analyzed during the current study will be made available through the National Institute of Mental Health’s Data Archive system, as well as by request from the primary investigator on reasonable request.

## Abbreviations

CAC: Children’s Advocacy Center
E3: Enhancing early engagement
E3w: Enhancing early engagement training—webinar only
E3w+c: Enhancing early engagement training—webinar plus consultation
EBP: Evidence-based practice
MI: Motivational interviewing
MDT: Multidisciplinary team
NCA: National Children’s Alliance
OMS: Outcome Management System
TIES: Training Intervention for the Engagement of Families

## Footnotes

1 q = number of variables
